# Supplementary material for: Attraction of Frankliniella occidentalis Females towards the Aggregation Pheromone Neryl (S)-2-Methylbutanoate and Kairomones in a Y-Olfactometer
Source: Insects. 2023 Jun 16;14(6):562. doi: 10.3390/insects14060562 (PMC10299560; doi:10.3390/insects14060562)
Supplement: Supplementary file 1 [file insects-14-00562-s001.zip › Figure S2.pdf]

```
###############################
####Quantification of SPE extracts
###############################


#packages
library(openxlsx)
library(effects)
library(investr)


###import data
setwd("C:\\Users\\charles.chappuis\\OneDrive - HESSO\\Documents\\Ra&D\\Lefort\\conf_publi")


ana <- read.xlsx("data_chappuis_2023.xlsx",sheet=3)
colnames(ana) <- c("name","rep","type","levels","istd","verb","meth","phero","panis")
str(ana)


###quantification
#values of blanks are neglected as they are very low. Headspace cells and cartridges were clean.


i=3
k=1


for(i in c(1:4)){
	prodi <- ana[,c(1,2,3,4,5,i+5)]
	nam <- names(prodi)[6]
		if(nam=="verb"){
		cali <- prodi[prodi$type=="Cal",]
		cali$levels <- cali$levels*0.992*0.94 #mass and purity taken into account
		cali$rel <- cali$verb/cali$istd
		mod <- lm(rel~levels,weight=1/levels,data=cali)
		summary(mod)
```

```
        }else{}

        if(nam=="meth"){
        cali <- prodi[prodi$type=="Cal",]
        cali$levels <- cali$levels*1.187*0.98
        cali$rel <- cali$meth/cali$istd
        mod <- lm(rel~levels,weight=1/levels,data=cali)
        summary(mod)
        }else{}

        if(nam=="phero"){
        cali <- prodi[prodi$type=="Cal",]
        cali$levels <- cali$levels*1.623*0.983
        cali$rel <- cali$phero/cali$istd
        calii <- cali[c(1:7),] #limit the calibration span, values above are useless
        mod <- lm(rel~levels+I(levels^2),weight=1/levels^2,data=calii)#
        summary(mod)
        }else{}

        if(nam=="panis"){
        cali <- prodi[prodi$type=="Cal",]
        cali$levels <- cali$levels*1.148*0.98
        cali$rel <- cali$panis/cali$istd
        mod <- lm(rel~levels,weight=1/levels,data=cali)
        summary(mod)
        }else{}
conc <- c()
for(k in c(1:nrow(prodi))){
        prodi$rel <- prodi[,6]/prodi[,5]
        xx <- prodi$rel[k]
        pred <- invest(mod,xx,interval="none",extendInt="yes")
```

```
                conci <- as.numeric(pred)
                conc <- c(conc,conci)
                }

        ana$pred <- conc
        colnames(ana)[9+i] <- paste(nam,"C",sep="")
}

ana
#write.table(ana,"quant_extracts_chappuis_2023.txt",sep="\t",col.names=T)

ana$verbCC <- 100*ana$verbC/(ana$levels*0.992*0.94)
ana$pheroCC <- 100*ana$pheroC/(ana$levels*1.623*0.983)
ana$methCC <- 100*ana$methC/(ana$levels*1.187*0.98)
ana$panisCC <- 100*ana$panisC/(ana$levels*1.148*0.98)

test <- ana$phero/ana$istd
(test+2.411e-04)/4.301e-05
```
