# Supplementary material for: Attraction of Frankliniella occidentalis Females towards the Aggregation Pheromone Neryl (S)-2-Methylbutanoate and Kairomones in a Y-Olfactometer
Source: Insects. 2023 Jun 16;14(6):562. doi: 10.3390/insects14060562 (PMC10299560; doi:10.3390/insects14060562)
Supplement: Supplementary file 1 [file insects-14-00562-s001.zip › Figure S3.pdf]

```r
#########################################
#R script to analyze data
#########################################

###packages required
library(ggplot2)
library(multcomp)
library(openxlsx)
library(reshape2)
library(dplyr)
library(scales)
library(ggh4x)
###set working directory
setwd("C:\\Users\\charles.chappuis\\OneDrive - HESSO\\Documents\\Ra&D\\Lefort\\conf_publi")



##########################################
#import data sets
##########################################

#Olfactometry
olf <- read.xlsx("Table_S1.xlsx",sheet=1)
str(olf)

#Analytical chemistry
ana <- read.xlsx("Table_S1.xlsx",sheet=2)
str(ana)

##########################################
#Analysis of olfactometry data
##########################################
```

```
###Shorten variables and compounds names

colnames(olf) <- c("comp","mass","repl","cont","treat","main","tot")

olf$comp <- ifelse(olf$comp=="methylisonicotinate","meth",ifelse(olf$comp=="p-anisaldehyde","panis",ifelse(olf$comp=="pheromone","phero","verb")))


###add log of mass and number of activated insects and percent of activated insects choosing treated arm (attraction)

olf$act <- olf$cont+olf$treat

olf$per <- 100*olf$treat/olf$act

olf$massL <- log10(olf$mass)

olf$cont+olf$treat


###Graph attraction as a function of compounds and quantity (mass)

mySum <- olf %>%

  group_by(comp,massL) %>%

        summarise(

        n=n(),

        mean=mean(per),

        sd=sd(per)

        )

mySum$stars <- c("","","**","","","**","","*","**","","","")# different from 50% probability, see below

str(mySum)


#need statsitical analysis below to fully graph with annotate function

p <- ggplot(mySum, aes(x=massL, y=mean, group=comp, color=comp)) +

        geom_line() +

        geom_point(size=2)+

        geom_segment(x=-0.2,y=50,xend=2.2,yend=50,linetype="dashed", color="black")+

        geom_errorbar(aes(ymin=mean-sd, ymax=mean+sd), width=0.1)+

        ylim(12.5,87.5)+

        #xlim(-0.2,2.2)+
```

```
	labs(x=expression(paste("Mass loaded [",mu,"g]")), y = "Attraction [%]")+
  scale_color_manual(values=c("blue","red","black","green"),name="",breaks=c("meth", "panis", "phero","verb"),labels = c("methyl isonicotinate", "p-anisaldehyde", "pheromone","verbenone"))+
	annotate("text",size=4,hjust=0,x=mySum$massL+0.05,y=mySum$mean+mySum$sd,label=mySum$stars,color=rep(c("blue","red","black","green"),each=3))+
	annotation_logticks(sides="b")
p+scale_x_continuous(breaks=c(0,log10(5),1,log10(50),2),labels=c(1,5,10,50,100),minor_breaks = c())
#ggsave("olf_mass_N.jpeg",width=1700,height=1100,device="jpeg",units="px",dpi="retina")



###Statistical analysis using GLM with binomial distribution and logit link function.
str(olf)
Tolf <- data.frame(prod=c(),repl=c(),ma=c(), att=c())
i="meth"
for(i in levels(as.factor(olf$comp))){
olfi <- olf[olf$comp==i,]
suA <- c(rep(1,sum(olfi$treat[olfi$mass==1])),rep(0,sum(olfi$cont[olfi$mass==1])))
replA <- c(rep(1,olfi$treat[olfi$mass==1&olfi$repl==1]),rep(2,olfi$treat[olfi$mass==1&olfi$repl==2]),rep(3,olfi$treat[olfi$mass==1&olfi$repl==3]),rep(1,olfi$cont[olfi$mass==1&olfi$repl==1]),rep(2,olfi$cont[olfi$mass==1&olfi$repl==2]),rep(3,olfi$cont[olfi$mass==1&olfi$repl==3]))
suB <- c(rep(1,sum(olfi$treat[olfi$mass==10])),rep(0,sum(olfi$cont[olfi$mass==10])))
replB <- c(rep(1,olfi$treat[olfi$mass==10&olfi$repl==1]),rep(2,olfi$treat[olfi$mass==10&olfi$repl==2]),rep(3,olfi$treat[olfi$mass==10&olfi$repl==3]),rep(1,olfi$cont[olfi$mass==10&olfi$repl==1]),rep(2,olfi$cont[olfi$mass==10&olfi$repl==2]),rep(3,olfi$cont[olfi$mass==10&olfi$repl==3]))
suC <- c(rep(1,sum(olfi$treat[olfi$mass==100])),rep(0,sum(olfi$cont[olfi$mass==100])))
replC <- c(rep(1,olfi$treat[olfi$mass==100&olfi$repl==1]),rep(2,olfi$treat[olfi$mass==100&olfi$repl==2]),rep(3,olfi$treat[olfi$mass==100&olfi$repl==3]),rep(1,olfi$cont[olfi$mass==100&olfi$repl==1]),rep(2,olfi$cont[olfi$mass==100&olfi$repl==2]),rep(3,olfi$cont[olfi$mass==100&olfi$repl==3]))
prodi <- rep(i,sum(c(length(suA),length(suB),length(suC))))
mai <- c(rep(1,length(suA)),rep(10,length(suB)),rep(100,length(suC)))
repli <- c(replA,replB,replC)
res <- data.frame(comp=prodi,repl=repli,ma=mai,att=c(suA,suB,suC))
```

```
Tolf <- rbind(Tolf,res)
}
str(Tolf)
Tolf$Cma <- as.factor(Tolf$ma)
Tolf$Cprod <- as.factor(Tolf$comp)
Tolf$Crepl <- as.factor(Tolf$repl)
mod <- glm(att~Cma*Cprod*Crepl,family=quasibinomial,data=Tolf)
summary(mod)

#effect of replicates
modB <- glm(att~Cma*Cprod+Crepl,family=quasibinomial,data=Tolf)
summary(modB)
anova(mod,modB,test = "Chisq")
modC <- glm(att~Cma*Cprod,family=quasibinomial,data=Tolf)
summary(modC)
anova(modB,modC,test = "Chisq") ###replicates has no significant effect, data can be pooled.

#relevel to determine the level of significance for data point
mod <- glm(att~relevel(Cma,"1")*relevel(Cprod,"verb"),family=quasibinomial,data=Tolf)
summary(mod)

mySum$stars <- c("","","**","","","**","","*","**","","","")# different from 50% probability



###Activation rates
mySumA <- olf %>%
  group_by(comp,massL) %>%
        summarise(
        n=n(),
        mean=mean(act),
```

```
	sd=sd(act)
	)

str(mySumA)


##########################################
#Analysis of release rates
##########################################

###Shorten variables and compounds names
colnames(ana) <- c("comp","repl","mass","conc","massE","rel","comment")
ana$comp <- ifelse(ana$comp=="methylisonicotinate","meth",ifelse(ana$comp=="p-
anisaldehyde","panis",ifelse(ana$comp=="pheromone","phero","verb")))

ana <- na.omit(ana)

###add log of mass
ana$massL <- log10(ana$mass)
ana$relL <- log10(ana$rel)
ana$compF <- as.factor(ana$comp)

###Graph release rate as a function of compounds and quantity (mass)
p <- ggplot(ana, aes(x=massL, y=relL, group=comp, color=comp)) +
	geom_point(size=2)+
	labs(x=expression(paste("Mass loaded [",mu,"g]")), y = "Release rate [ng/min]")+
	scale_color_manual(values=c("blue","red","black","green"),name="",breaks=c("meth",
"panis", "phero","verb"),labels = c("methyl isonicotinate", "p-anisaldehyde",
"pheromone","verbenone"))+
	theme(legend.text=element_text(size=8))+
	#xlim(0,2)+
```

```
annotate("text",size=3,x=c(rep(1.75,4)),y=c(2.89,2.68,2.38,1.6),label=c("a","a","b","c"),color=c("blue","green","red","black"))+

geom_abline(intercept=c(0.943,0.445,-0.402,0.925),slope=c(rep(1.052,4)),color=c("blue","red","black","green"))+#parameters from linear model below

annotation_logticks(sides="bl")+

scale_x_continuous(breaks=c(0,log10(5),1,log10(50),2),labels=c(1,5,10,50,100),minor_breaks = c())+

scale_y_continuous(breaks=c(log10(0.5),0,log10(5),1,log10(50),2,log10(500),3),labels=c(0.5,1,5,10,50,100,500,1000),minor_breaks = c())


p


#ggsave("releaserate_mass_N.jpeg",width=1700,height=1100,device="jpeg",units="px",dpi="retina")


###stastistical analysis with linear models

mod <- lm(relL~massL*compF,data=ana)

summary(mod) #no interaction terms significant, slopes are not significantly different among the compounds


modN <- lm(relL~massL+compF,data=ana)

summary(modN)

anova(mod, modN)


modNN <- lm(relL~massL,data=ana)

summary(modNN)

anova(modN, modNN)


#relevels

modN <- lm(relL~massL+relevel(compF,"phero"),data=ana)

summary(modN)


10^c(0.939,0.925,0.445,-0.402)#retransformed data to obtain ng/min for the intercept
```

```
################################################
#Analysis of release rates and behavioral data
################################################

anaS <- ana %>%
  group_by(comp,massL) %>%
  summarise(
    n=n(),
    mean=mean(relL, na.rm=T),
    sd=sd(relL, na.rm=T)
  )

str(anaS)
10^c(-0.352,0.609,1.68)
10^c(2.09,-0.352)
10^c(-0.352,3,2.61)
anaS$attr <- mySum$mean
anaS$attrSD <- mySum$sd
anaS$stars <- mySum$stars

p <- ggplot(anaS, aes(x=mean, y=attr, group=comp, color=comp)) +
        geom_line() +
        geom_point(size=2)+
        geom_segment(x=-1,y=50,xend=3.5,yend=50,linetype="dashed", color="black")+
        geom_errorbar(aes(ymin=attr-attrSD, ymax=attr+attrSD), width=0.2)+
        ylim(12.5,87.5)+
        #xlim(-0.5,3.5)+
        labs(x=expression("Release rate [ng/min]"), y = "Attraction [%]")+
   scale_color_manual(values=c("blue","red","black","green"),name="",breaks=c("meth", "panis", "phero","verb"),labels = c("methyl isonicotinate", "p-anisaldehyde", "pheromone","verbenone"))+
```

```
	theme(legend.text=element_text(size=8),axis.text.x = element_text(size = 6),)+
	annotate("text",size=4,hjust=0,x=anaS$mean+0.1,y=anaS$attr+anaS$attrSD,label=anaS$stars,
color=rep(c("blue","red","black","green"),each=3))+
	annotation_logticks(sides="b")+
	scale_x_continuous(breaks=c(log10(0.5),0,log10(5),1,log10(50),2,log10(500),3),labels=c(0.5,1
,5,10,50,100,500,1000),minor_breaks = c())

p
ggsave("olfactometer_releaserate_N.jpeg",width=1700,height=1100,device="jpeg",units="px",dpi="r
etina")

anaS$Rmean <- 10^anaS$mean
anaS$gPhase <- anaS$Rmean/0.15
anaS$test <- anaS$Rmean/122



##################################
#P-value that a proportion is significantly different from 50% with a pool of 75 thrips
###################################

Nthrips <- 75
i=1
pval <- data.frame(cat=c(),val=c())
for(i in c(1:(Nthrips-1))){
	veca <- c(rep(1,i),rep(0,Nthrips-i))
	cata <- rep(i,Nthrips)
	vecb <- c(rep(1,i+1),rep(0,Nthrips-(i+1)))
	catb <- rep(i+1,Nthrips)
	ro <- data.frame(cat=c(cata,catb),resp=c(veca,vecb))
	ro$cat <- as.factor(ro$cat)
	mod <- glm(resp~cat, family=quasibinomial, data=ro)
```

```
        sumM <- summary(mod)
        pv <- coef(sumM)[7]
        pvali <- data.frame(cat=i,val=pv)
        pval <- rbind(pval,pvali)
}

pval$prop <- 100*pval$cat/Nthrips

p <- ggplot(pval, aes(x=prop, y=val)) +
        geom_line() +
        geom_point(size=1)+
        geom_segment(x=0,y=0.05,xend=100,yend=0.05,linetype="dashed",color="black")+
        labs(x="Attraction [%]", y="Pvalue")+
        scale_x_continuous(breaks = seq(0,100,12.5),labels = seq(0,100,12.5))
p
ggsave("stat_problem.jpeg",width=1400,height=1000,device="jpeg",units="px",dpi="retina")
```
