# Supplementary figures and images for: Attraction of Frankliniella occidentalis Females towards the Aggregation Pheromone Neryl (S)-2-Methylbutanoate and Kairomones in a Y-Olfactometer
Source: Insects. 2023 Jun 16;14(6):562. doi: 10.3390/insects14060562 (PMC10299560; doi:10.3390/insects14060562)

Figure S1 : results of the NMR analysis of the synthesized pheromone, neryl (S)-2-methylbutanoate

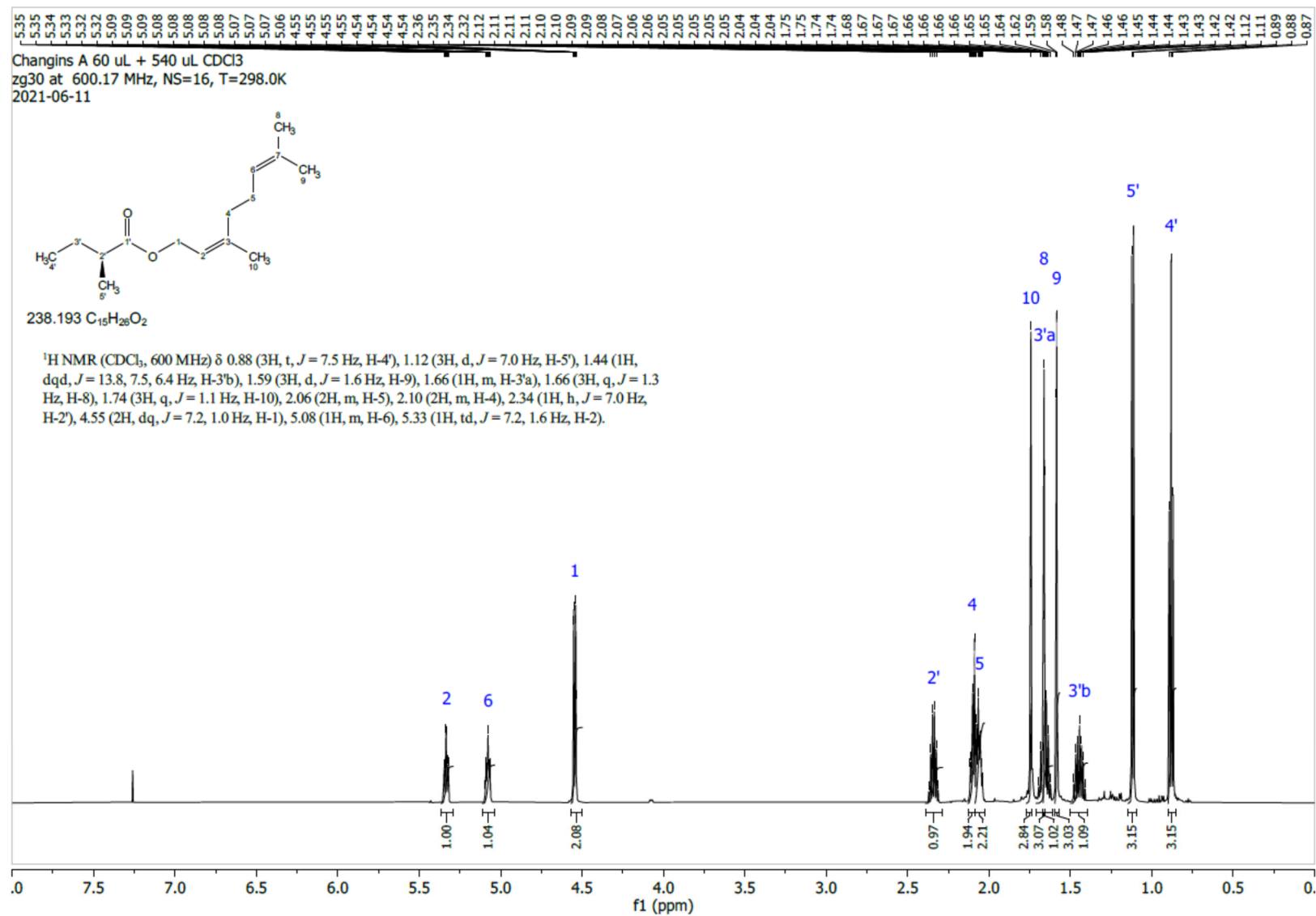

e.

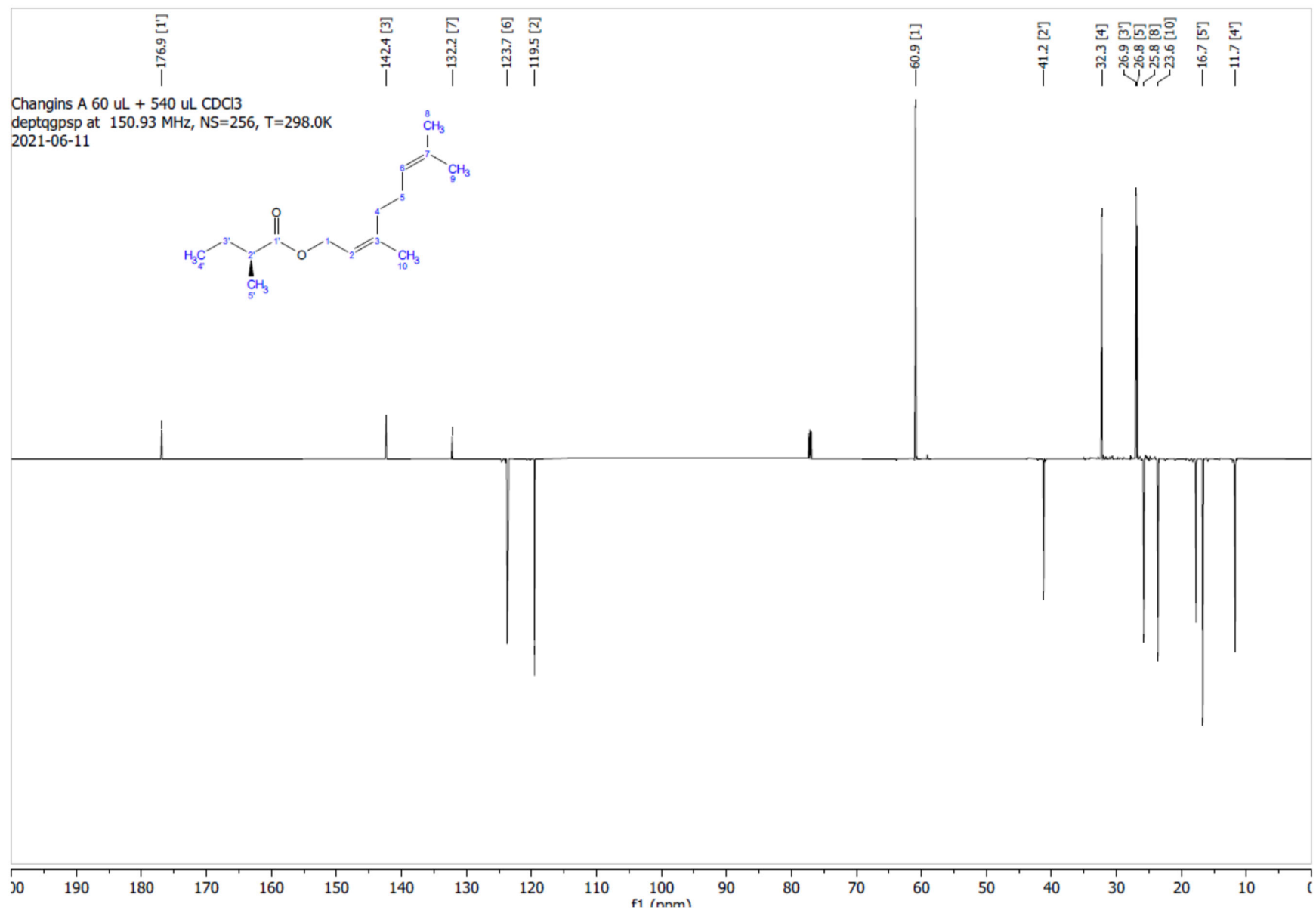

Supplement: Supplementary file 1 [file insects-14-00562-s001.zip › Figure S1.pdf]
